# First-generation merozoites of caprine *Eimeria christenseni* are capable to invade and egress primary host endothelial cells *in vitro*

**DOI:** 10.3389/fvets.2025.1717848

**Published:** 2025-12-10

**Authors:** L.M.R. Silva, A. Ruiz, E. Barba, S. Lopéz-Osorio, J.A. Molina, J.M. Molina, A. Taubert, C. Hermosilla

**Affiliations:** 1 Faculty of Veterinary Medicine, Institute of Parasitology, Justus Liebig University Giessen, Giessen, Germany; 2 Egas Moniz Center for Interdisciplinary Research (CiiEM), Egas Moniz School of Health & Science, Caparica, Portugal; 3 Faculty of Veterinary Medicine, Department of Animal Pathology, University of Las Palmas de Gran Canaria, Arucas, Spain; 4 Faculty of Agrarian Sciences, CIBAV Research Group, Veterinary Medicine School, University of Antioquia, Medellín, Colombia

**Keywords:** *Eimeria christenseni*, merozoite I, primary endothelial cells, host cell active invasion, host cell egress

## Abstract

The caprine *Eimeria christenseni* species belongs to the phylogenetic clade of pathogenic ruminant *Eimeria,* replicating within the endothelial cells of central lymph capillaries of the ileum villi *in vivo*. Investigations on *E. christenseni*-host endothelial cell interactions, including cell invasion, egress, apoptosis, senescence, cell cycle, cytoskeleton, cell metabolism and endothelium-derived innate immune reactions are possible to achieve through permissive *in vitro* culture systems. Therefore, we here established a suitable *in vitro E. christenseni* (strain GC) culture system using primary bovine umbilical vein endothelial cells (BUVEC) for the development of first-generation macromeronts. After 18–22 days post infection (p.i.), the intracellular sporozoites matured into fully developed *E. christenseni*-macromeronts, releasing viable merozoites I. Interestingly, two different types of *E. christenseni*-merozoites I were observed, i.e., thinner and thicker merozoites I. The thinner ones were more active, presented typical gliding motility, and were found intracellularly shortly after their release, while the thicker ones were less active and invasive to BUVEC. Thinner *E. christenseni* merozoites I actively invaded and egressed host cells by breaching the plasma membrane without host cell lysis, a phenomenon exclusively reported so far for apicomplexan sporozoites of *Plasmodium yoelii* and *Eimeria bovis*. Additionally, intracellular *E. christenseni* merozoites I were monitored over time (up to 30 days), thereby revealing no further development into meront II stages. Further research is needed to assess whether primary endothelial cells of caprine origin could support the complete life cycle of *E. christenseni in vitro*. This novel *in vitro* system will contribute not only for further studies on *Eimeria-*derived invasion- and egress strategies, endothelial cell-derived innate immune reactions, but also for merozoites I- and antigen production requested for vaccination strategies as already reported for other ruminant *Eimeria* species.

## 1 Introduction

Caprine coccidiosis is a widely recognized and geographically widespread parasitic disease, causing either catarrhal or haemorrhagic diarrhoea, dehydration and weight loss primarily in goat kids and also in adult animals (1–7). Given the global population of over one billion goats reared in 2023 (8), caprine coccidiosis exerts a significant impact on profitability of goat industry. The consequences of this enteric disease include substantial costs associated with treatments, compromised growth rates, diminished milk yields, and, in severe cases, mortality among younger animals (9). As a result, effective management and control strategies for caprine coccidiosis are of paramount importance in guaranteeing the wellbeing and economic viability of goat farming operations worldwide (10).

Among the 16 identified caprine *Eimeria* species, *Eimeria ninakohlyakimovae*, *Eimeria arloingi,* and *Eimeria christenseni* belong phylogenetically to the group of ruminant *Eimeria* replicating within endothelial cells of the central lymph capillaries of the ileum villi *in vivo* (11, 12). Despite replicating in highly immunoreactive host endothelial cells, these species undergo a massive intracellular replication resulting in macromeronts (>200 to 400 μm in size) during their first merogony phase *in vivo* (12, 13). During this long-lasting macromeront development of 18–22 days, endothelial cells suffer extreme morphological and metabolic changes *in vitro* and *in vivo*, including significant cytoskeleton- and nuclear changes (11, 14), cholesterol metabolism (15), and senescence (16). The nucleus of infected host endothelial cells carrying *Eimeria*-macromeronts changes from heterochromatin content to a so-called “fried-egg”-shaped nucleus containing euchromatin and a nucleolus coalescing to form single or multiple nucleoli, as reported not only for *Eimeria bovis* and *Eimeria zuernii* in cattle (16–18) but also for caprine *E. ninakohlyakimovae* and *E. arloingi* (9, 12, 19). These common replication features strongly suggest shared evolutionary history for *Eimeria* species proliferating in highly immunoreactive endothelium, producing hundreds of thousands of first-generation merozoites (syn. merozoites I). In line, it has been speculated that sporozoites of a common ancestor within the subphylum Apicomplexa were capable to successfully breach cell membranes of intestinal epithelial cells to migrate deeper achieving either final endothelial cell- or hepatic-cell invasion *in vivo* as reported for *E. ninakohlyakimovae* (20, 21). This unique invasive capacity of sporozoites has previous been described exclusively for *E. bovis* (13) and *Plasmodium yoelii* (22). In case of *E. bovis*, sporozoites rapidly invade and egress from host endothelial cells by breaching cell membranes without cell lysis to seek for the ideal host cell to develop further into macromeronts (13). Similarly, *P. yoelli* sporozoites traverse hepatocytes for achieving macromeront development (22). These apicomplexan sporozoites use an alternative invasion mode by breaching the plasma membrane followed by rapid cell membrane repair, i.e., host cells survive this process, and without forming a typical parasitophorous vacuole (PV) (13, 22). Thus, both sporozoite species encounter intricate challenges as they surmount physical barriers to gain access to their final target host cells to perpetuate life cycles within the host organisms. Nonetheless, the exact route followed by *E. bovis*- and *P. yoelii* sporozoites *in vivo* to reach respective target host cells remains elusive.

The energetic costs of migrating sporozoites alone as well as generating thousands of merozoites I are enormous and rely on scavenging host cell metabolism (15, 23–26). Moreover, cholesterol auxotrophic *Eimeria* sporozoites cannot provide all lipids and nutrients for massive merozoite I production, and the parasite needs to scavenge lipids from parasitized endothelial cells. Thus, colonization of endothelial cells might represent a niche within ruminant small intestine for scavenging cholesterol and/or other lipids. Consistently, *Eimeria* sporozoite endothelium invasion results in efficient modulation of *de novo* cholesterol biosynthesis, low-density lipoprotein (LDL)-mediated uptake during macromeront formation (15, 27, 28). Consequently, cellular cholesterol was altered in *E. bovis*-infected endothelial cells as indicated by the up-regulation of cholesterol-25-hydrolase and sterol O-acyltransferase (28). Similarly, internalization of all LDL modifications, i.e., LDL, oxidized LDL (oxLDL), acetylated (acLDL), and the lipoprotein-oxidized receptor 1 (LOX-1) expression (15) was increased in *Eimeria* macromeront carrying endothelial cells. To continue investigations on cholesterol metabolism, amino acid/nucleotide biosynthesis, glycolysis and glutaminolysis in *Eimeria* species infecting endothelial cells, reliable *in vitro* cell systems are mandatory (29), including organoids (24) as well as *ex vivo* intestinal explants (26).

Up until recently, only two pure caprine *Eimeria* species were kept worldwide for basic research purposes, i.e., *E. ninakohlyakimovae* (GC strain) (9) and *E. arloingi* (two strains: strain A from Portugal and other strain from Iran) (19, 30). Hence, efforts to gain access to new goat *Eimeria* species infecting intestinal endothelial cells was achieved by a new field-isolated *E. christenseni* [strain Gran Canaria (GC)] from Canary Island, Spain (31). Interestingly, this *E. christenseni* isolate showed lower pathogenicity in contrast to other caprine *Eimeria* species sharing the same endogenous niche of development, i.e., infecting and developing the first schizogony in endothelial cells (9, 19, 30). The possibility to compare *Eimeria* species with different pathogenic potential developing in such highly immunoreactive host cells could be of high interest to perform basic research focused on investigating the mechanisms of pathogenicity.

We here describe a permissive *in vitro* system for the new field-isolated *E. christenseni* (strain GC) from Canary Island, Spain (31). This *in vitro* culture system is based on primary bovine umbilical vein endothelial cells (BUVEC) as host cells to optimize macromeront formation, aiming to replicate *in vitro* its *in vivo* conditions as closely as possible. For the first time, freshly-released and highly motile thin merozoites I formed in *E. christenseni* macromeronts actively invaded and egressed host endothelial cells *in vitro*, using an alternative mode of invasion increasing understanding of apicomplexan invasion/egress strategies. Additionally, this *in vitro* system will expectantly serve as useful tool for further research on survival strategies of *E. christenseni* stages (i.e., sporozoites, trophozoites and merozoites I), nutrient requirements, host cell-derived immune reactions, as well as, for testing novel anticoccidial drugs.

## 2 Materials and methods

### 2.1 Ethical statement

All animal procedures were carried out in strict accordance with national ethics, current European legislation on animal welfare (ART13TFEU), and protocols approved by the institutional review board (OEBA-ULPGC 15/2019R1).

### 2.2 Animals

The animals used in this study were sourced from a local goat farm located in Canary Island, Spain, which specializes in rearing the Majorera breed. For conducting experimental *E. christenseni* (strain GC) infections, three goat kids (1–3 days old) were individually identified with ear tags and subsequently transferred to the Experimental Animal House at the Faculty of Veterinary Medicine, University of Las Palmas de Gran Canaria, Spain. Upon arrival, the animals underwent washing and drying procedures to remove any potential adhered oocysts. Following this procedure, the goat kids were placed in steel metabolic boxes that had previously been thoroughly cleaned, disinfected by flame, prior to their allocation as reported elsewhere (9).

On the day of arrival, goat kids received a single dose of diclazuril [1 mg/kg body weight (BW); Rumicox®, Elanco] and a seven-day course of halofuginone (0.1 mg/kg BW; Halocur®, Intervet) treatment. Access to the housing stable containing metabolic boxes of goat kids was restricted to exclusively authorized personnel, and strict biosecurity measures were adhered to, aiming to prevent any coccidian infection. To maintain a high hygiene standard, all personnel entering the animal area wore clear clothing and utilized new protective gear, such as sterile gloves, caps, and shoe covers, on every visit. For the initial 2 weeks of age, goat kids were nourished with a milk replacer (Bacilactol®, Capisa) and gradually introduced to commercial pellets (Starting Concentrate®, Capisa) thereafter. Sterilized water and hay were given *ad libitum*.

### 2.3 Parasites

To obtain the novel *E. christenseni* strain (GC), individual faecal samples were collected from naturally infected goat kids at a local farm of Canary Island as described previously (31). For oocyst replication, three goat kids (*n* = 3) aged 3 weeks old were experimentally infected with 2 × 10^5^ *E. christenseni* sporulated oocysts to assure parasite endogenous replication. Prior to oral infection, oocysts underwent three washes with distilled water to eliminate any traces of potassium dichromate. From 21 to 28 days p. i., faecal samples were collected from trays allocated under the metabolic cages.

For oocyst isolation, the faeces were mixed with regular tap water at a 1:1 ratio and passed through sieves with decreasing pore diameter, down to 100 μm. The washed faecal solution was mixed with a saturated sugar solution (density 1.3 g/L) at a 1:1 ratio and floated onto glass slides, which were rinsed with distilled water every 2 h for three consecutive days. Subsequently, each faecal-distilled water mixture was centrifuged at 2,300 × *g* for 20 min, and the supernatant was discarded. The resulting sediment was diluted 1:1 once more with distilled water in a glass flask. The obtained oocysts were then suspended in a 2% potassium dichromate solution (*v*/*v*) and transferred to Erlenmeyer bottles. To facilitate sporulation this process was performed under constant aeration using an aquarium pump for 7 days at room temperature (RT). The mixed potassium dichromate-oocysts sediment was stored in vented cap cell culture flasks at 4 °C until further *in vitro* experiments which were carried out at the Institute of Parasitology of the Justus Liebig University Giessen, Giessen, Germany.

### 2.4 *Eimeria christenseni* oocyst excystation

*Eimeria christenseni* oocyst excystation protocol was carried out following a previously described and improved procedure (32). In brief, the sporulated oocyst suspension (7.5 × 10^5^ oocysts/mL) underwent centrifugation at 600 × *g* for 12 min to eliminate potassium dichromate traces. The resulting oocyst-containing pellet was then resuspended in a 4% (*v*/*v*) sodium hypochlorite working solution (Roth) and magnetically stirred on ice for 20 min to remove possible debris adhering to oocyst walls. Subsequently, the oocysts were transferred to 50 mL plastic tubes (Sarstedt), mixed by vortex for 15 s, and centrifuged again (300 × *g*, 5 min) to remove larger debris particles. The oocysts-containing supernatant was mixed with distilled water (1:1) and pelleted (600 × *g*, 15 min). The resulting pellet containing the oocysts was then diluted in distilled water (10–20 mL) and passed through a 70 μm filter (PluriSelect) for a final debris elimination. Tap water (40 mL) was poured through the filter to clean up for possible stacked oocysts. This oocyst suspension was subsequently filtered with a 20 μm filter (PluriSelect) and washed with 50 mL of distilled water to remove small particles, oocysts were aimed to be retained in the filter. By inverting the filter and passing distilled water through the back opening, all oocysts were recovered. This step was repeatedly performed to assure maximal recovering (up to 40–50 mL were obtained). Finally, filtered oocysts were centrifuged (600 × *g,* 15 min), re-suspended in a fresh sterile filtered (0.2-μm filter, Sarstedt) 0.02 M l-cysteine HCl-H_2_O/0.2 M NaHCO_3_ working solution (*w*/*v*) (Merk) and incubated for 20 h at 37 °C in a 100% CO_2_ atmosphere in a T75 cell culture flask (closed cap, Sarstedt). After incubation, oocysts underwent a final centrifugation step (600 × *g*, 15 min) and the resulting pellet was re-suspended in sterile filtered (0.2-μm filter, Sarstedt) excystation solution [Hank’s balanced salt solution (HBSS, Gibco) supplemented with 0.4% (*w*/*v*) trypsin (Sigma-Aldrich) and 8% (*v*/*v*) bovine bile (obtained fresh from a local slaughter every 3–4 weeks and kept at −20 °C)] up to 3 h at 37 °C in a 5% CO_2_ atmosphere. The excystation process was hourly monitored using an inverted microscope (IX81, Olympus) equipped with a digital camera (XM10, Olympus). Free sporozoites were collected by centrifugation (600 × *g*, 15 min) and filtered (20-μm filter; PluriSelect) to remove any remaining oocysts and sporocysts. Sporozoites were finally washed twice with cell culture media [mod ECGM: ECGM medium (Promocell®) with M199 (Sigma-Aldrich)] at a ratio of 1:3, supplemented with 500 U/mL penicillin (Sigma-Aldrich), 50 μg/mL streptomycin (Sigma-Aldrich) and 5% FCS (foetal calf serum; Biochrom), and counted in a Neubauer chamber before being used for infection assays.

### 2.5 Primary host endothelial cells

Primary bovine umbilical vein endothelial cells (BUVEC) were isolated as previously described (33). Three different BUVEC isolates were cultured at 37 °C and 5% CO_2_ atmosphere in modified endothelial cell growth medium (modECGM). BUVEC of less than three passages were used for all studies. BUVEC isolates (*n* = 3) were seeded in T25 cell tissue culture flasks (Greiner) or in 12-well plates (Sarstedt), with 1 × 10^4^ cells per cm^2^.

### 2.6 First merogony of *Eimeria christenseni in vitro*

BUVEC were seeded into T25 tissue culture flasks (Greiner) and incubated at 37 °C and 5% CO_2_ atmosphere. The medium (modECGM; 4 mL) was replaced every 2–3 days until the cultures reached (80%–90%) confluence, resulting in BUVEC monolayers prepared in triplicate (*n* = 3). Then, 2.5 × 10^5^ freshly excysted *E. christenseni* sporozoites were added per cell culture flask. Culture medium was changed at 24 h p. i. to remove extracellular sporozoites and then successively every 2 days. To follow *E. christenseni* intracellular development, infected host cells were checked daily under an inverted microscope (IX81, Olympus) equipped with a digital camera (XM10, Olympus) for a period of 24 days p. i. Infection rates were determined in five power vision fields randomly selected at 400 × magnification at 24 h p. i. after first medium change, using the software CellSens® Dimension 1.7® (Olympus). Given that glucose supplementation was beneficial for the *in vitro* development of the closely related caprine species, *E. arloingi* (19) in the same model, here we supplemented modECGM with 10 mM glucose (Sigma-Aldrich) throughout the merogonic replication.

### 2.7 Alternative invasion and egress capacity of *Eimeria christenseni* merozoites I in primary endothelial cells

To evaluate in detail the ability of free *E. christenseni* merozoites I to invade and egress endothelial cells shortly after their release from mature macromeronts (from day 18 p. i. onwards), a top-stage incubator (IBIDI®) was used to maintain temperature, humidity, and CO_2_ levels similar to standard cell culture incubator parameters previously described. Motile *E. christenseni* merozoites I were followed under an inverted microscope (IX81, Olympus) equipped with a digital camera (XM10, Olympus), and images were acquired every 75 ms over time using the software CellSens® Dimension 1.7® (Olympus). The resulting video was extracted in “.avi format.” Single frames of specific times were obtained with the software CellSens® Dimension 1.7® (Olympus). Imaging post-processing was carried out in Image J® using minor adjustments of brightness and contrast and montage plugins.

Given that this was the first time such merozoites I features were documented in any caprine or ruminant *Eimeria* species and that invasion/egress was documented in the same monolayer, merozoites I were collected (600 × *g*, 10 min) and, thereafter, added to non-infected BUVEC (1.5 × 10^5^ per well—12-well plate) to understand if these processes would also take place without the presence of macromeronts. *E. christenseni* merozoites I capacity of invasion/egress of new BUVEC and further develop into macromeronts was followed over time (18 days p. i.) as previously described.

## 3 Results

### 3.1 Excystation of *Eimeria christenseni* sporulated oocysts

After excystation of sporulated *E. christenseni* oocysts and to isolate viable sporozoites, a novel cleaning process based on different filters was used (32) and validated for caprine *Eimeria* species. Without using complicated gradients with expensive reagents and expensive ultracentrifuges, it was possible to separate *E. christenseni* oocysts from most of faecal debris. Even if it was not feasible to eliminate debris completely, especially small particles (<10 μm), almost pure oocysts could later be incubated in the excystation medium. Due to the lack of caprine bile availability, which would better resemble the *in vivo* situation, bovine bile was included in the excystation medium. This excystation medium mimics the *in vivo* situation in terms of ruminant bile, its enzymes, salts and CO_2_ atmospheric pressure, allowing proper isolation of viable *E. christenseni* sporozoites. As early as 40 min of incubation, free sporozoites were observed in the excystation medium. After 2.5–3 h of incubation, free-released sporozoites were highly motile and presented typical gliding and contractility movements. Active sporozoites were released either from free sporocysts (34) or directly from the sporocyst into the oocyst circumplasm and later evading the oocyst through the micropyle to the medium (19). In the latter, the oocyst wall was not degraded but was distinctly thinner. Before release, sporozoites became very active in the oocyst cavity until reaching the micropyle and egressed. Only fully sporulated oocysts were observed with a thinner wall after l-cysteine incubation in 100% CO_2_. On the contrary, non-sporulated oocysts remained with visible intact wall. A total of 70%–80% of the sporozoites were released from fully sporulated and activated oocysts. *E. christenseni* sporozoites presented typical gliding motility, visible refractile bodies as observed in sporozoites of other ruminant *Eimeria* species, confirming viability.

### 3.2 Intracellular *Eimeria christenseni* sporozoite development into macromeronts

**Figure 1:**
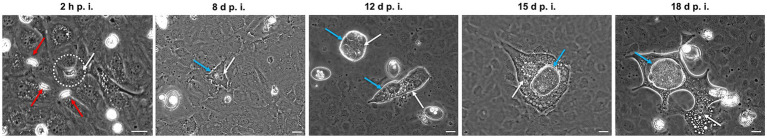
Development of *Eimeria christenseni* immature macromeronts in BUVEC, 8–18 days post infection (d p. i.). *E. christenseni* sporozoites were observed intracellularly as soon as 2 h p. i. (white circle), while others remained extracellularly (red arrows). From 8 d p. i. nuclei of infected BUVEC presented “fried-egg” shape (white arrows) like other ruminant endothelial *Eimeria* spp. infections. At 8 d p. i., a small immature meront (blue arrow) is observed very close to the host cell nucleus. Meronts presented different phenotypes (rounded or elongated) (12 d p. i., blue arrows) sometimes with multiple chambers (12 d p. i.) or presenting a single chamber (15 and 18 d p. i., blue arrow). Scale bar 20 μm.

After *E. christenseni* sporozoites were seeded on BUVEC monolayers, they remained active showing the same movements above-mentioned on cellular surface. At 2 h p. i., the first intracellular sporozoites were documented (Figure 1, white circle), while the majority of the sporozoites were still extracellular (Figure 1, red arrows). In general, intracellular *E. christenseni* sporozoites were found close to the nucleus (Figure 1, white arrows) of infected endothelial cells. Most infected host cells carried a single sporozoite, and seldom infections with two or more sporozoites per cell were observed. The mean infection rate at 24 h p. i. was 8.30% ± 1.31% (mean ± standard deviation). From 4 d p. i. onwards, intracellular trophozoites increased their size, becoming immature meronts. Macromeront development continued as expected. From 8 d p. i., infected BUVEC presented characteristic “fried-egg” shape host cell nucleus (Figure 1, white arrows), that persisted until the end of macromeront development. Additionally, small trophozoites (Figure 1, 8 d p. i., blue arrow) and immature meronts were observed close to host cell nuclei. Most macromeronts (Figure 1, 12–18 d p. i., blue arrows) were flat (Figure 1, 12 d p. i.) or rounded (Figure 1, 15 and 18 d p. i.), and presented multiple (Figure 1, 12 d. p. i.) or single chamber arrangement (Figure 1, 15 and 18 d p. i.). Some infected BUVEC presented vacuolization, showing that massive parasite proliferation was indeed a high stress inducer to this host cell type. Note mentioning, macromeront development in the presence of 10 mM glucose was similar to control and no improvement in size was observed (data not shown). During macromeront formation, some *E. christenseni* sporozoites were observed extracellularly after egressing from previously infected BUVEC.

### 3.3 *Eimeria christenseni* merozoites I present different phenotypes

**Figure 2:**
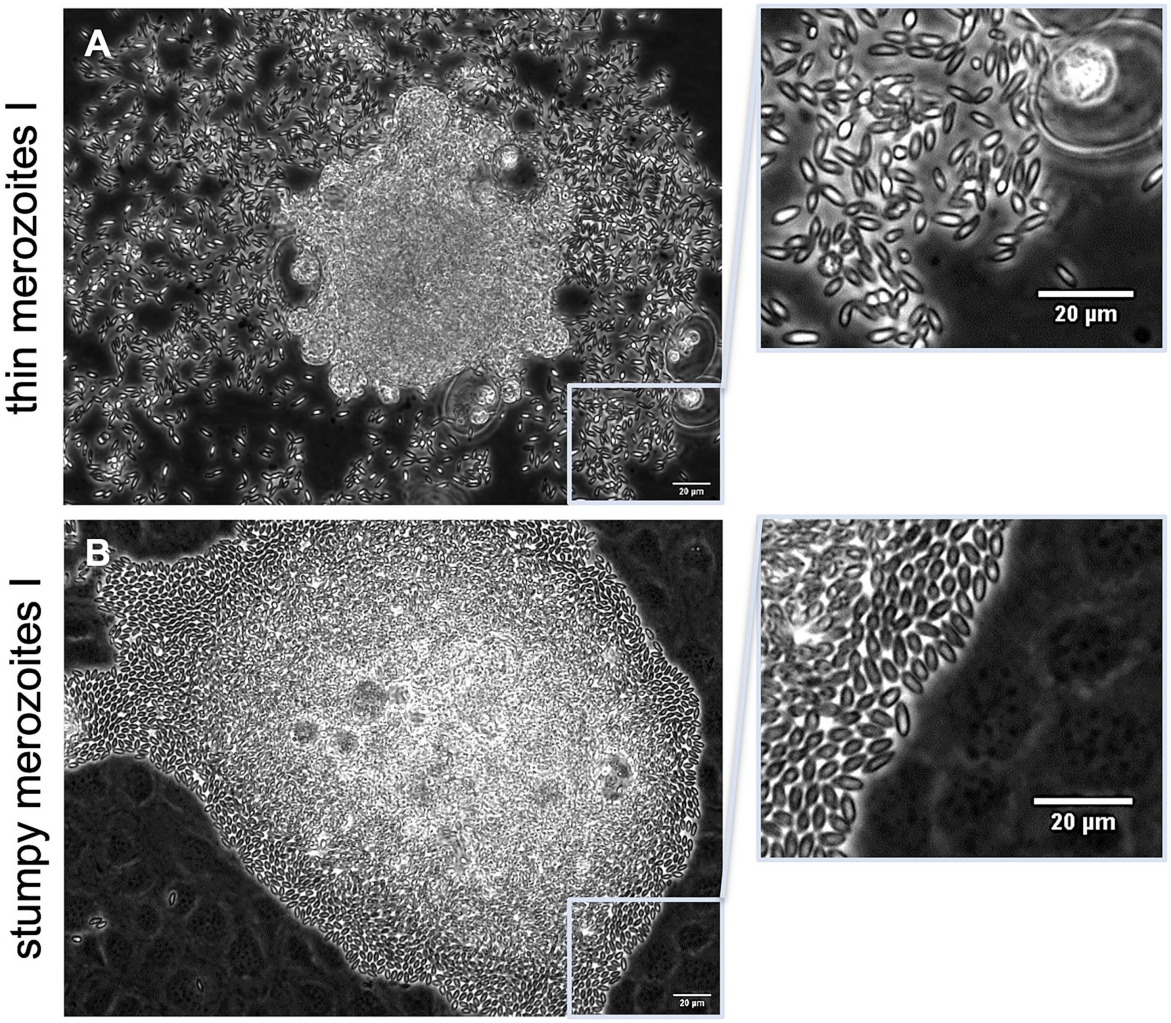
Mature *Eimeria christenseni* macromeronts presented fully developed merozoites I (18–22 d p. i.). Two phenotypes of merozoites I were released **(A)** thinner and more active/motile merozoites, which moved away from ruptured macromeront, and **(B)** thicker, stumpy and less active merozoites, which remained clustered after the rupture of macromeronts. Scale bar 20 μm.

Mature *E. christenseni* macromeronts presented fully developed merozoites I that were released from day 18 p. i. onwards. Even though all merozoites I presented similar sizes, two phenotypes of *E. christenseni* merozoites I were registered here (Figure 2). Most common observed phenotype was characterized by thinner and highly active and motile merozoites I (Figure 2A) that usually were found gliding away from ruptured macromeronts, appearing disperse in the monolayer, a feature that is needed *in vivo* for the completion of the second merogony in epithelial cells of the large intestine. This phenotype represents the characteristic merozoite I observed for other studied ruminant *Eimeria* species in cattle, e. g. *E. bovis*. A second type of merozoites I were also identified. These stages presented a more bulky and thicker structure, being less active and therefore remaining clustered around the recently ruptured macromeront (Figure 2B). At first visualization, they were mistaken as not viable merozoites I but with closer observation, it was possible to verify that they were also motile and viable. While thin merozoites I presented constant movement with extensive gliding motility, stumpy merozoites I seemed to be longer in close contact with each other and were found less disperse in the monolayers. Surprisingly, both types of *E. christenseni* merozoites I were also observed when cultures were supplemented with 10 mM glucose and in the same proportion (circa 20%–30%), confirming that this might be a species-specific feature not related with viability or suitability of the *in vitro* system.

### 3.4 *Eimeria christenseni* merozoites I are able to actively invade and egress host endothelial cells without losing their vitality and motility

**Figure 3:**
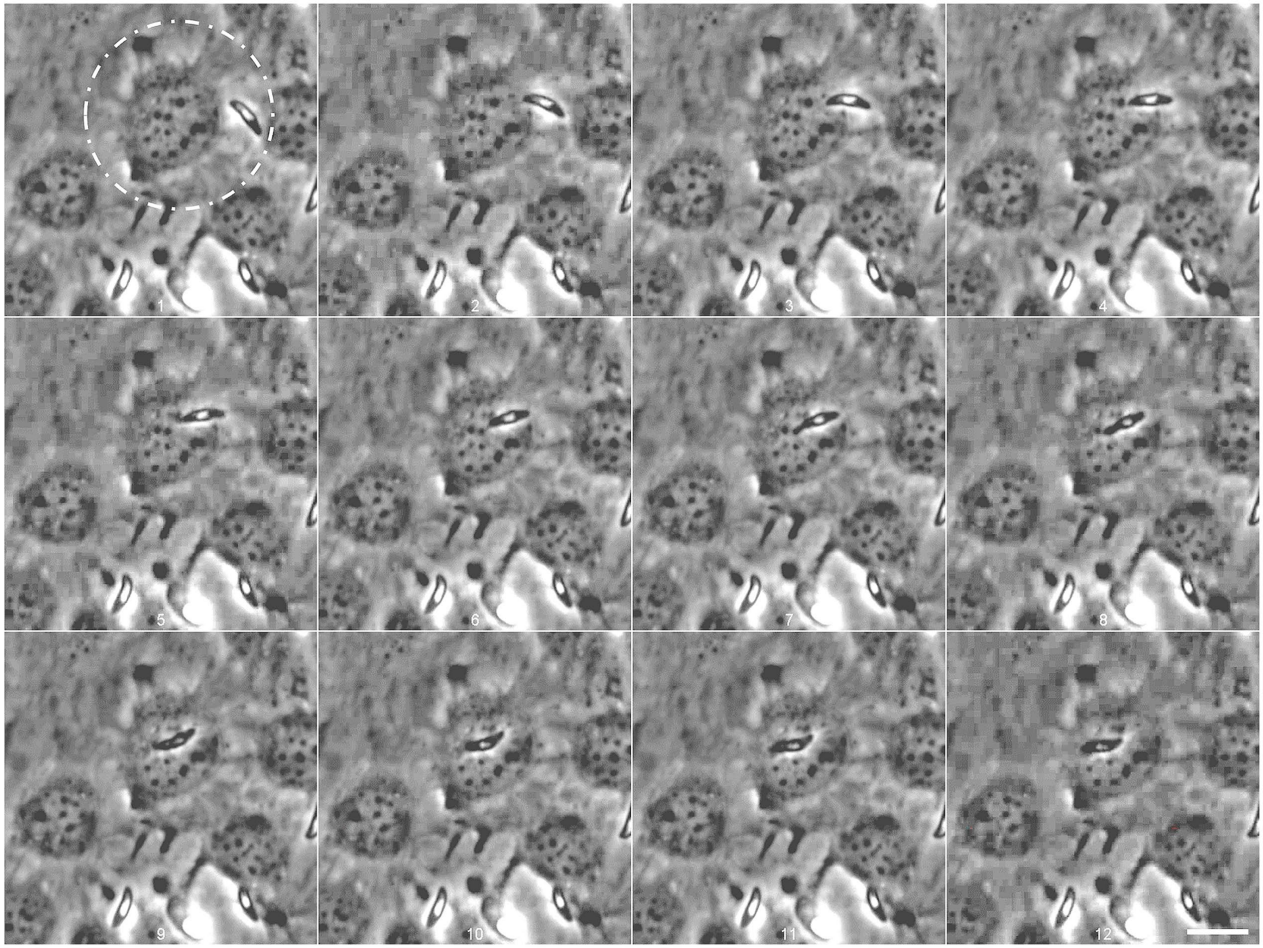
*Eimeria christenseni* merozoites I actively invaded BUVEC shortly after release from the macromeront (white circle). Motile merozoites I approached BUVEC close to the nucleus (1–2) and started active invasion (3) that was completed within 6 s (2–9). The merozoite I remained intracellularly after minor adjustments (9–12) for over 12 min. Images were acquired every 750 ms. Scale bar 20 μm.

**Figure 4:**
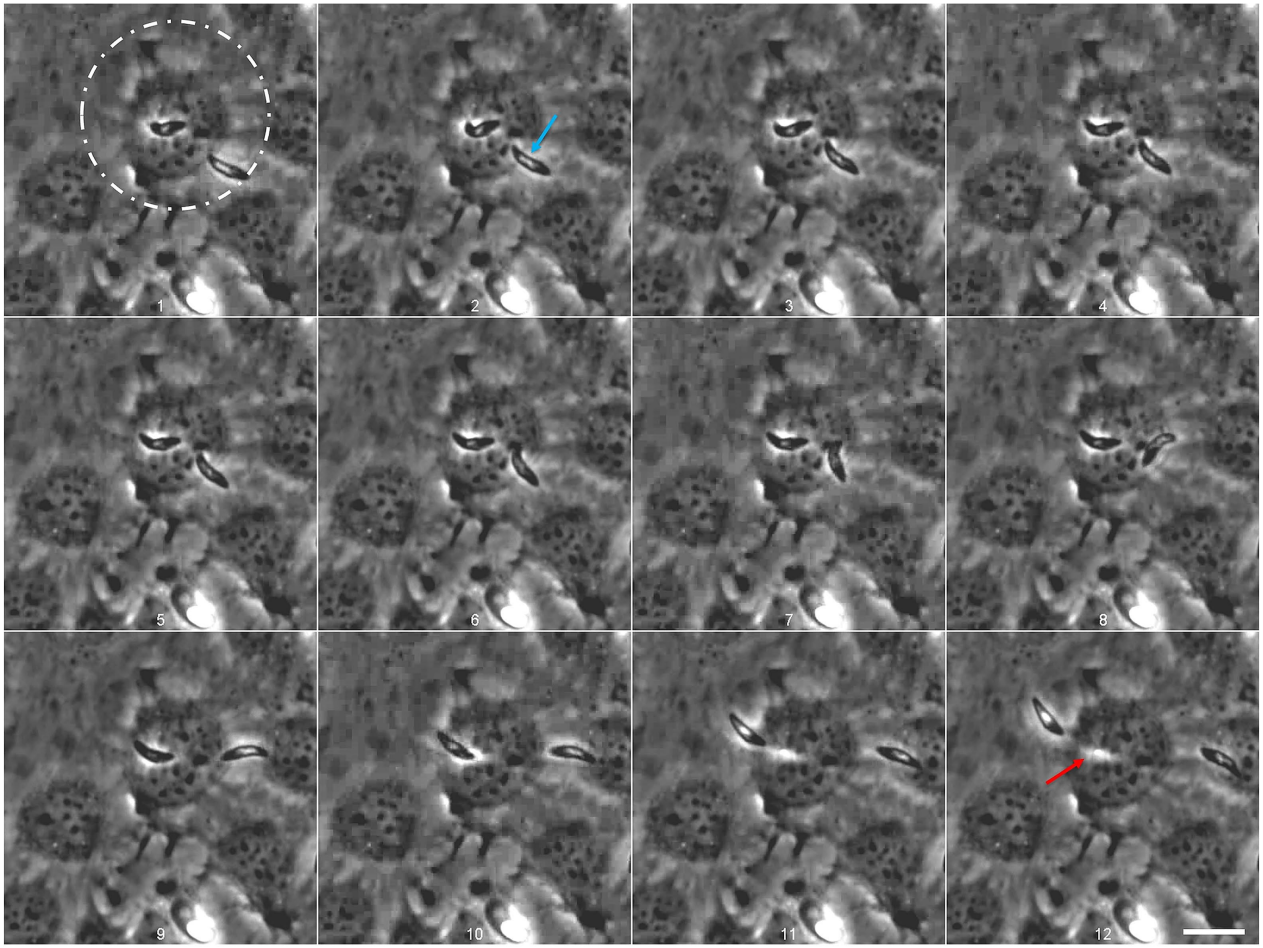
*Eimeria christenseni* merozoite I attempts to invade an already infected host cell. The merozoite I, that was observed intracellularly for more than 12 min, located above the nucleus, started to move after another merozoite I tried to invade the same host cell (1). Merozoite I egress was completed after 13 s since the first movement (here the last 9 s are depicted: 1–12). After host cell egress, a white track (12: red arrow), resembling a “footprint” of the merozoite I shape, can be recognized in previously invaded BUVEC. The second merozoite (2: blue arrow) failed to invade the same host cell (9). Scale bar 20 μm. Images were acquired every 750 ms. Scale bar 20 μm.

For the first time, we observed *Eimeria* merozoites I actively invading (Figure 3; Supplementary video S1) and egressing (Figure 4; Supplementary video S2) host endothelial cells without compromising their vitality. Freshly released *E. christenseni* merozoites I presented well-known gliding motility over BUVEC monolayers. Some of these merozoites I initiated attempts to invade BUVEC by gliding above the cell first at 2 h p. i. (Figure 3, 1–6, white circle). With its apical end, the merozoite I penetrated the surface of host cell (Figure 3, 7–8) by breaching the plasma membrane—a constriction of the merozoite shape was clearly visible while it passes through the membrane (please refer to Supplementary video S1). In only 6 s, merozoite I invasion was completed (Figure 3, 4–9), but the formation of a PV was not perceived. Intracellular merozoite I was located very closely to the nucleus, but this new feature was observed exclusively with the thinner merozoite I phenotype, equally in the control and the glucose-supplemented groups. Interestingly, merozoites I were observed attempting to invade exclusively non-infected cells, or cells already containing other merozoites I, but not cells infected with a non-developed sporozoite (most likely as a “dormozoite”—or “hypnozoite” stage).

Most merozoites I that invaded host cells remained intracellularly for several minutes but eventually egressed. Other merozoites I were observed already intracellularly but it was not possible to determine for how long these stages remained inside host cells. The merozoite I followed in Figure 3, remained 12 min intracellularly. At this point, the merozoite I initiated egress (Figure 4, white circle; Supplementary video S2). Remarkably, another merozoite I attempted to invade the same cell (Figure 4, 1–5, blue arrow) indicating that some cells might present higher permissiveness than others. A second merozoite I failed to invade the cell and glided away (Figure 4, 6–12). At the same time, the first merozoite I started moving slowly (Figure 4, 1–5) and its movement intensified (Figure 4, 9) and culminated with the egress of the cell (Figure 4, 9–12; Supplementary video S2). After complete egress, a white track (Figure 4, 12: red arrow), resembling a “footprint” of the merozoite shape, could be recognized in the previously invaded host cell, which did not show any deformation or leakage after egress process. Noteworthy, recently egressed merozoites I continued to present gliding movements on the cellular monolayer, indicating that breaching into the cell, remaining intracellularly and breaching out of the cell was not detrimental for their survival. Until day 24 p. i., no further development of intracellular merozoites I was documented, including in the glucose-supplemented medium experimental group.

### 3.5 *Eimeria christenseni* merozoites I neither invade naïve host cells nor develop intracellularly into second meronts

**Figure 5:**
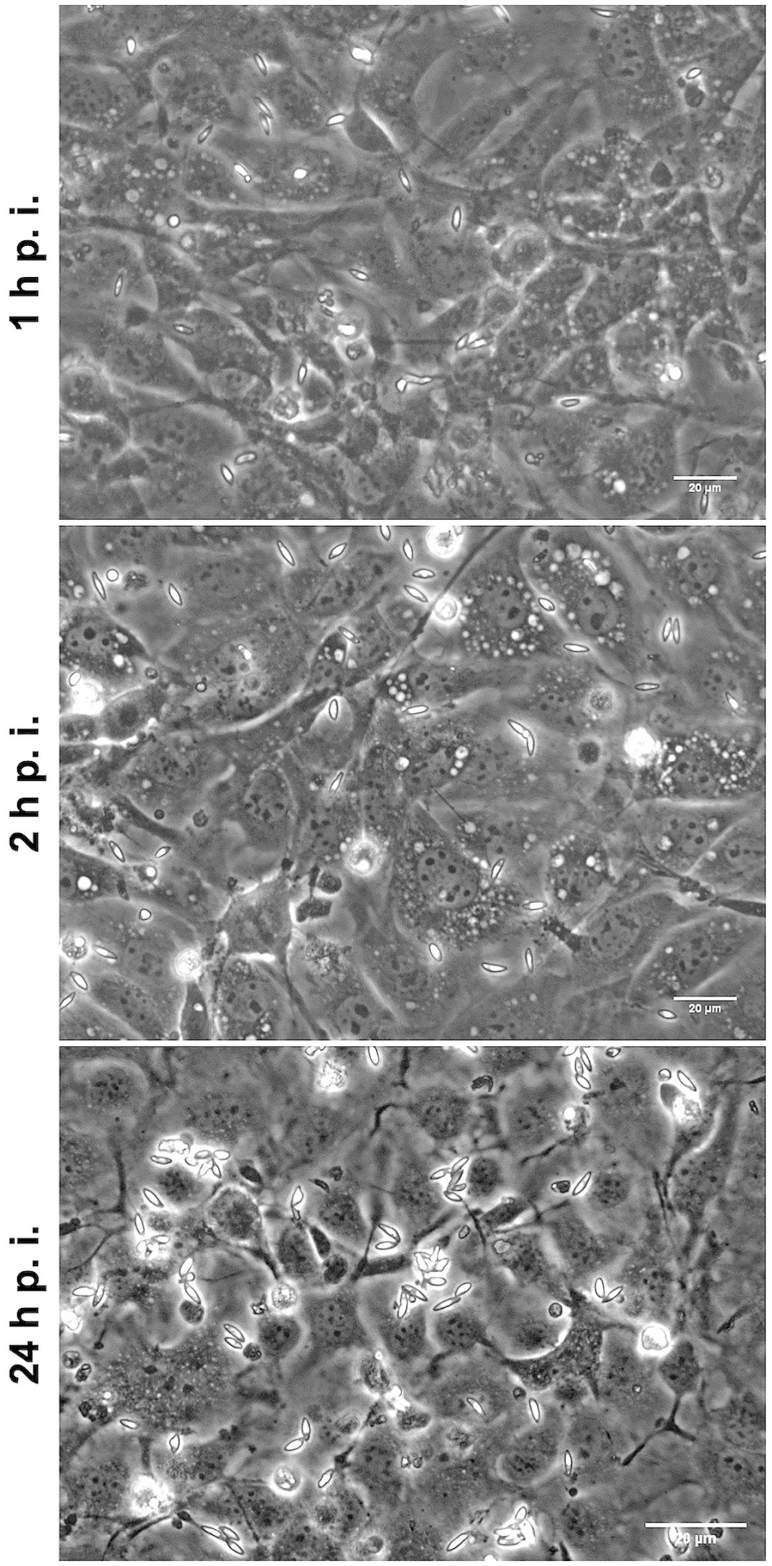
*Eimeria christenseni* merozoites I were collected from the supernatant of infected monolayers and seeded into new non-infected BUVEC. At 1- and 2-h p. i., merozoites I were viable and presented typical gliding and contractile motilities. At these time points, no merozoites I were observed intracellularly. At 24-h p. i., merozoites I were less active, some were dead, and none of them were found intracellularly.

To better understand if *E. christenseni* merozoite I breaching ability was also possible in naïve BUVEC, i.e., without previous contact to *E. christenseni* stages (sporozoites, sporocysts, oocysts, oocyst circumplasm), merozoites I were collected, washed and, thereafter, seeded in these naïve BUVEC monolayers. After 1 h and 2 h of exposure, no merozoites I were found intracellularly (Figure 5). Most parasites remained viable and presented typical gliding motility being fairly distributed on the monolayer. At 24 h p. i., some merozoites I were still active and vital but others (40%–50%) were observed darker, started to round up and were immotile (Figure 5, 24 h p. i.). No infected/invaded cells were ever observed, indicating that such behaviour might be dependent on paracrine effects derived from other *E. christenseni*-infected host cells. The same results were obtained in three independent assays, proving that this phenomenon was independent of host and individual donor variations, but rather dependent on the inability of these parasite stages to invade naïve host cells.

## 4 Discussion

The well-known 3R concept in science, i.e., replacement, refinement, and reduction of animal experiments, has been largely followed over the last years (35). Thus, suitable *in vitro* systems allowing the total replacement of animal experimentation have been developed for other closely related ruminant *Eimeria* species [*E. bovis* (36), *E. zuernii* (18) (bovine), *E. arloingi* (19), *E. ninakohlyakimovae* (34) (caprine) and *Eimeria ovinoidalis* (37) (ovine)]. By implying primary host endothelial cell systems, which represent the closest system to the *in vivo* situation (11), a great number of investigations have been carried out. Therefore, we here present a permissive *in vitro* culture system, based on primary bovine endothelial cells for the novel caprine *E. christenseni* (strain GC). Given that caprine umbilical vein endothelial cells (CUVEC) could be contaminated with *Mycoplasma* spp. and that treatment with Plasmocin™ (Invitrogen) (34) has a detrimental effect on intracellular development of *Eimeria* spp., CUVEC were not included in the experiment.

The first step to achieve a successful *Eimeria* spp. *in vitro* system is the access to viable and infective sporozoites. Therefore, an excystation medium that mimics the *in vivo* situation containing ruminant bile, enzymes, salts, and adequate CO_2_ atmospheric pressure was applied, thereby allowing proper isolation of viable *E. christenseni* sporozoites. As previously reported for excystation protocols for other caprine coccidian species (*E. ninakohlyakimovae* and *E. arloingi*) (19, 34) and ovine species (*E. ovinoidalis*) (37), bovine bile was added to the excystation medium instead of caprine bile, due to unavailability. Shortly after the beginning of the incubation in excystation medium (40 min), sporozoites of *E. christenseni* were free. Sporozoite egress from sporulated oocysts either followed *E. bovis* (32) and *E. ninakohlyakimovae* (34) process, in which oocyst wall ruptures and activated sporozoites egress from free sporocysts, or sporozoites became activated inside sporocysts still contained in oocysts, resembling *E. arloingi* excystation process (19), in which sporozoites egress the sporocyst into the oocyst circumplasm and later on evade the oocyst through the micropyle to the medium, unveiling high motility in both the cases.

Freshly released sporozoites presented typical gliding motility and were allowed to invade host endothelial cells *in vitro*. Contrary to other caprine *Eimeria* parasitizing intestinal epithelial cells, sporozoites of *E. christenseni* must replicate within endothelial cells of the central lymph capillaries of lacteals *in vivo*, corresponding to the molecular phylogenetic group of ruminant *Eimeria* species forming large macromeronts (200–400 μm). It is postulated that sporozoites of a common ancestor species were able to migrate deeper into new host tissues to infect lymph endothelium, thereby colonizing a new niche in small intestine of ruminants *in vivo* (12), possibly to fulfil specific nutritional and developmental requirements as recently demonstrated for *E. bovis* (15, 17, 27, 28). This endothelium-based system is formed by primary bovine umbilical endothelial cells that were found permissive to *E. christenseni* sporozoite invasion with further development into trophozoites at 4 d p. i. Immature meronts (8 d p. i.) and large macromeronts containing massive numbers of viable merozoites I were also observed. The first merogony with macromeront formation might be one of the most crucial steps of ruminant *Eimeria* life cycle and being able to complete this peculiar development on highly immunoreactive host endothelial cells must be a challenging event (11, 17, 34). As previously mentioned, endothelial cells are highly reactive cells that are accessible only to extremely specific or specialized apicomplexan sporozoites. Moreover, energetic costs of migration and production of massive offspring (>140.000 merozoites I) are only possible if the parasite can modulate the host cell at different levels and additionally scavenge host cell metabolism (11). Therefore, implementation of new *in vitro* systems to investigate parasite-derived modulation of host cells is crucial to develop and test effective anticoccidial drugs against pathogenic ruminant *Eimeria* species.

For the first time, *Eimeria* merozoites I were observed actively invading endothelial cells *in vitro*, showing a similar invasion mechanism as seen for sporozoites (33, 34). These *E. christenseni* merozoites I remained temporarily intracellularly until they egressed host endothelial cells, without signs of decreased vitality and cell death. Until now, none of the other ruminant *Eimeria* species studied in the past, i.e., *E. bovis*, *E. zuernii, E. ninakohlyakimovae*, *E. arloingi,* and *E. ovinoidalis* in the same *in vitro* cell system, presented such merozoite I ability. This egress behaviour has exclusively been reported for sporozoites of apicomplexan *E. bovis* and *P. yoelli* which are able to breach the plasma membrane of cells without formation of a parasitophorous vacuole (PV) (13, 22). Particularly for *Plasmodium* sporozoites, after infection of hepatocytes, some stages were observed in the cytosol of host cells, both *in vivo* and *in vitro*, without a PV (38). This alternative mechanism of invasion was associated with the ability of sporozoites to traverse several cells before identifying a cell in which they decide or are allowed to develop further (13, 38). The traverse ability might trigger signaling pathways required for inducing invasion through PV formation or might allow sporozoites to identify necessary metabolic properties of host cells for adequate macromeront development (13, 38). As such, it was recently reported that in the presence of low host endothelial-derived cellular glycolytic activities, simultaneous egress of *E. bovis* sporozoites was achieved (16). After the egress of *E. christenseni* merozoites I, the previously invaded host cell seemed to recover rapidly and no leakage could be observed, showing that the passage of merozoites I was not immediately detrimental to host cells. The same breaching feature was recognized when *E. bovis* sporozoites crossed the plasma membrane of BUVEC without forming a PV (13). On the contrary, not all hepatocytes survived active *P. yoelii* sporozoite passages, as some of them presented leakage of cytosolic material to the medium, followed by cell death, while others were able to rapidly reseal the plasma membrane (22).

After egress of BUVEC, merozoites I seemed viable, active and motile, therefore, they were exposed to naïve BUVEC monolayers to let them invade new cells. Nevertheless, merozoites I failed to invade naïve BUVEC showing that invasion might be related to paracrine signaling originated from previously infected host cells [e.g., release of exosomes, extracellular vesicles (EV)], or even macromeront components after rupture that might influence the merozoite I ability to actively invade primary endothelial cells. A more detailed investigation on this question could identify difficulties encountered in developing the second merogony in cell culture systems (33, 34) and further contribute to achieve the complete *in vitro* development of ruminant *Eimeria* species.

*Eimeria christenseni* first merogony occurs in highly immunoreactive endothelial cells, resembling other highly pathogenic species, i.e., *E. bovis* and *E. zuernii* (cattle), *E. ninakohlyakimovae* and *E. arloingi* (goats), *E. ovinoidalis* (sheep). However, recent evidence suggests that *E. christenseni* GC strain might be less pathogenic than other close related caprine *Eimeria* species replicating in endothelial cells *in vivo* (31). Therefore, access to a strain that retains endothelial replication capacity but exhibits reduced pathogenicity provides a valuable model for comparative studies with highly pathogenic species. Additionally, its *in vitro* excystation and development generate different stage-specific antigens (oocysts, sporocysts, sporozoites, trophozoites, and merozoites I), creating new opportunities for molecular analyses and vaccination studies as previously reported for chicken, rabbits, and ruminants (39–42).

We report the first successful *in vitro* culture of *E. christenseni* GC in primary bovine endothelial cells, recording merozoite I invasion and egress for the first time in a ruminant *Eimeria*. This system demonstrates that *E. christenseni* shares key developmental features with other endothelial-replicating species while displaying reduced pathogenicity, offering a unique comparative model. By providing access to sequential developmental stages, it opens new opportunities for molecular characterization, host-parasite interaction studies, and antigen discovery. Beyond its scientific value, this model advances 3R principles and provides a platform for developing next-generation anticoccidial resources, including vaccination strategies.

## Data Availability

The original contributions presented in the study are included in the article/Supplementary material, further inquiries can be directed to the corresponding authors.

## Supplementary material

SUPPLEMENTARY VIDEO S1 BUVEC being actively invaded by *Eimeria christenseni* CG strain merozoite I.

SUPPLEMENTARY VIDEO S2 *Eimeria christenseni* CG strain merozoite I egress of BUVEC.
